# From Logs to Bags: A Metabolic Blueprint of Sanghuang Cultivation Revealed by UPLC-Q-TOF-MS/MS and Amino Acid Profiling

**DOI:** 10.3390/molecules30132829

**Published:** 2025-07-01

**Authors:** Kefan Xu, Lingli Chen, Chenchen Wu, Haiyang Wang, Fei Wu, Jingzhe Pu, Yazhong Zhang

**Affiliations:** 1 School of Pharmacy, Anhui University of Chinese Medicine, Hefei 230012, China; kefanxu735@gmail.com (K.X.); 15255831553@163.com (C.W.); 2 Anhui Institute for Food and Drug Inspection and Research, Hefei 230051, China; chenll0421@126.com; 3 School of Pharmaceutical Sciences, Anhui Medical University, Hefei 230031, China; haiyangchz@163.com (H.W.); 18355639960@163.com (F.W.)

**Keywords:** Sanghuang, amino acid, metabolite, bioactivity, antioxidant

## Abstract

Sanghuang (SH), a natural fungal resource used for food and medicinal purposes, has drawn considerable attention due to its pharmacological effects and efficacy. This study focused on Wild Sanghuang (WS) and Sanghuang cultivated using two different methods: Duanmu Sanghuang and Mycelium Sanghuang. Using UPLC-O-TOF-MS, we conducted an in-depth analysis of the secondary metabolites present in SH. The content of 18 amino acids was measured using an automated amino acid analyzer. The results demonstrated major differences in secondary metabolites, including flavonoids, organic acids, amino acids, and their derivatives, between WS and cultivated Sanghuang (CS). The total amino acid content in WS surpassed that of CS, with segmental trunk SH exhibiting a total amino acid content 1.3 times (*p* < 0.05) greater than that of bag material SH. This variation may be linked to the biosynthetic pathways of valine, leucine, isoleucine, and flavonoids. By comparing the metabolomic and amino acidomic differences between WS and artificially CS, this study aims to provide a scientific basis for understanding the nutritional and medicinal value of various cultivation methods for SH and offer theoretical support for the future development of SH-related products.

## 1. Introduction

In traditional Chinese medicine, the relationship between food and medicine, as well as their synergistic effects on health maintenance and disease intervention, is highly valued. The notion that “food tonics are superior to medicinal tonics” [1,2] illustrates the concept of the shared origin of food and medicine. SH is a medicinal fungus with substantial research and development value. It primarily parasitizes the trunks of mulberry trees, characterized by their yellow-brown fruiting bodies, which is reflected in its name. The active compounds in SH not only exhibit antioxidant [3] and immune-enhancing [4] properties but also effectively inhibit the production of inflammatory factors [5], demonstrating potential for anti-tumor effects [6], blood sugar reduction [7], and uric acid level lowering [8]. Recently, the rising demand for functional foods has prompted a gradual exploration and application of various active ingredients from SH in food processing. The beta-glucans, triterpenoids, and various amino acids abundant in SH possess high nutritional value and exhibit bioactivity [9]. However, due to its slow natural growth rate [10] and the increasing demand for Wild SH (WS), existing wild resources are insufficient to meet market needs.

Since the 1990s, cultivated Sanghuang (CS) has drawn considerable attention. Although numerous varieties of SH exist, two primary cultivation methods have emerged as the most successful: Duanmu Sanghuang (DS) cultivation, which involves burying SH mycelium in specific segments of wood within outdoor shade sheds [11,12], and Mycelium Sanghuang (MS) cultivation, which uses wheat grains and wood shavings as substrates [13,14]. The following provides a detailed overview of these two cultivation methods. The DS cultivation method entails inoculating SH mycelium onto specific segments of wood and cultivating it in outdoor shade sheds. This method benefits from the natural growth habits of SH, fostering its growth under suitable environmental conditions. It has several advantages, including strong adaptability to various climatic conditions, high yield potential, vigorous mycelial growth, and notable flavor and medicinal value. Conversely, the MS cultivation method uses wheat grains and wood shavings as substrates. After high-temperature sterilization, the SH mycelium is inoculated. This method’s advantages include the rapid cultivation of large quantities of SH, a short cultivation cycle, quick results, and the ability to enhance yield and quality by controlling substrate composition. It is particularly suitable for large-scale production due to its ease of management and control.

Both cultivation methods offer distinct advantages and disadvantages. The DS method aligns with the natural ecology of SH, making it suitable for small-scale or household cultivation. In contrast, the MS method is better suited for large-scale production, facilitating rapid and higher yields. The choice of cultivation method depends on specific planting conditions, market demand, and personal experience. With ongoing technological advancements, further improvements and innovations in SH cultivation methods are anticipated in the future. This study aimed to explore the differences in amino acid composition of SH under different cultivation methods and its relationship with secondary metabolites. WS and two CS varieties were selected as research subjects, and comparative metabolomics methods were employed to determine and analyze the amino acid composition and content of SH. Additionally, a systematic metabolomics analysis of secondary metabolites in SH was conducted, aiming to provide a scientific basis and reference for the selection of quality cultivation methods, quality evaluation, and the development and utilization of SH. By investigating the influence of various cultivation methods on amino acids and secondary metabolites of SH, this study not only enriches the existing knowledge of SH but also offers new perspectives and ideas for its applications in medicine and food.

## 2. Results

### 2.1. Characteristic Analysis of Secondary Metabolites in SH Samples

**Figure 1:**
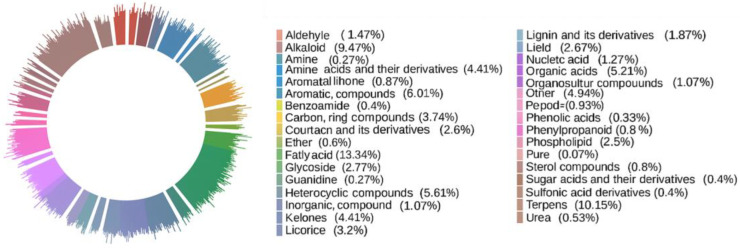
Primary classification pie chart of secondary metabolites cultivated in three different ways.

To investigate the metabolomic characteristics of SH samples, 28 batches of small-molecule metabolites were analyzed using UPLC-Q-TOF-MS. Comparison with the HMDB database revealed that the metabolites could be classified into the following categories: 100 Flavones, 76 Terpenes, 45 Aromatic Compounds, 42 Heterocyclic Compounds, 40 Phenols, 39 Organic Acids, 37 Others, 33 Amino Acids and their derivatives, 33 Ketones, 28 Carbon Ring Compounds, 26 Alkaloids, 25 Fatty Acids, 24 Licorices, 21 Phospholipids, 21 Coumarin and its derivatives, 20 Lipids, 17 Glycosides, 14 Nucleic Acids, 14 Lignin and its derivatives, 11 Aldehydes, eight Inorganic Compounds, eight Organosulfur Compounds, seven Phenolic Acids, seven Peptides, six Phenylpropanoids, six Sterol Compounds, six Quinones, six Ethers, five Pures, five Anthraquinones, four Ureas, three Benesulfonamides, three Benzoamides, three Sugar Acids and their derivatives, two Amines, two Guanidines and two Sulfonic Acid derivatives. Figure 1 shows the primary classification of secondary metabolites of Sanghuang cultivated in three different ways. Through an in-depth analysis of 749 metabolites, we further explored their biological activities and potential medicinal value.

### 2.2. Analysis of Metabolite Variations Between Wild and Cultivated SH

**Figure 2:**
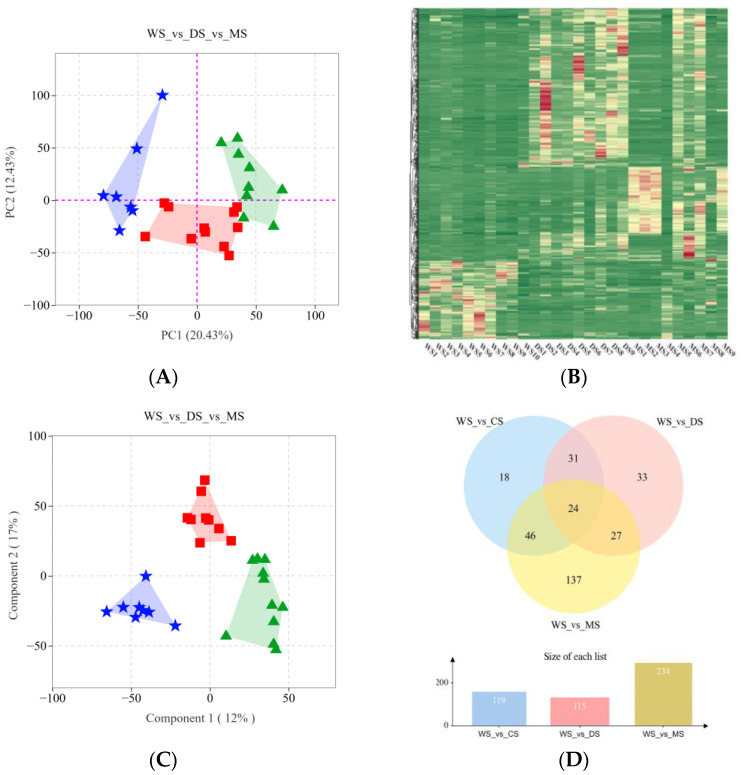
Metabolomic analysis of wild and cultivated Sanghuang; (**A**) Principal component analysis of Sanghuang metabolite data: ★ indicates MS, ▲ indicates WS, and ■ indicates DS; (**B**) WS_vs_DS_vs_MS Hierarchical clustering heatmap; (**C**) OPLS-DA plot for WS, MS, and DS metabolite data: ★ indicates MS, ▲ indicates WS, and ■ indicates DS; (**D**) Venn diagram illustrating pairwise comparisons of “WS_vs_CS,” “WS_vs_DS,” and “WS_vs_MS”.

To analyze the metabolic profiles of wild and CS samples, PCA and HCA were performed using 749 identified metabolites. As shown in Figure 2A, the PCA results revealed notable metabolic differences and variations among the samples. WS, DS, and MS samples were distinctly separated along principal component 1 and principal component 2, which accounted for contribution rates of 20.43% and 12.43%, respectively, totaling 32.86%. The clear differentiation among the WS, MS, and DS sample groups indicated substantial disparities in their metabolic profiles. The HCA heatmap (Figure 2B) further corroborated these findings, demonstrating distinct clustering of SH samples based on different cultivation methods, with noticeable metabolic differences among WS, DS, and MS. Samples within the same group clustered closely together, reinforcing the reliability of the sample classification.

To comprehensively compare the metabolic differences between WS and these two CS, we used the OPLS-DA paired comparison method to analyze the data of “WS vs. CS”, “WS vs. DS”, and “WS vs. MS”. The results showed that the OPLS-DA model had a high degree of fit, with Q2 > 0.86, R2Y > 0.99 (*p* < 0.05). In pairwise comparisons, by selecting log2Fold ≥ 2 (upregulated) or log2Fold ≤ 0.5 (downregulated) and VIP value ≥ 1, we found 157, 109, and 267 differential metabolites in the comparisons of “WS vs. CS”, “WS vs. DS”, and “WS vs. MS”, respectively. The Venn diagram (Figure 2D) showed that there were 46 differential metabolites shared by the three cultivation methods of SH. These metabolites are closely related to the geographical origins of different cultivation methods and are influenced by climatic factors, which may serve as potential biomarkers for inferring the geographical origins of WS and CS.

As the Venn diagram shows, in the comparison between “WS and MS,” 85 differential metabolites were identified, which included two organic oxygen-containing compounds, one tannin, 16 other substances, nine terpenes, one saponin, two phenols, 13 flavonoids, nine organic acids, two alkaloids and their derivatives, three organic hydroxy compounds, three lactones, one phenylpropane and polyketones, three lignans and coumarins, four alcohols, four glycosides, two quinones, two benzene ring compounds, two amino acids and their derivatives, two organic phosphate compounds, two lipids and lipid-like molecules, and two organic heterocyclic compounds. In the comparison between “WS and DS,” 87 differential metabolites were identified, encompassing two organic oxygen-containing compounds, one tannin, 16 other substances, 10 terpenes, one saponin, two phenols, 13 flavonoids, nine organic acids, three alkaloids and their derivatives, three organic hydroxy compounds, three lactones, one phenylpropane and polyketones, three lignans and coumarins, four alcohols, four glycosides, two quinones, two benzene ring compounds, two amino acids and their derivatives, two organic phosphate compounds, two lipids and lipid-like molecules, and two organic heterocyclic compounds.

It is important to note that among the 46 differential metabolites, WS exhibited higher levels of three flavonoids and three amino acids along with their derivatives compared to DS and MS, while DS and MS demonstrated more prominent levels of one lipid and one alkaloid. These findings suggest that wild environments may be more conducive to the accumulation of amino acids and their derivatives, as well as flavonoids in SH, while inhibiting the production of lipids and alkaloids. Further analysis of the biological functions of these differential metabolites may reveal the potential medicinal value of SH. Flavonoids have garnered considerable attention due to their biological activities, including antioxidant, anti-inflammatory, and anti-tumor effects. This is particularly true in traditional Chinese medicine, where flavonoids are considered important components for enhancing immune function and improving cardiovascular health [15]. Consequently, the higher content of flavonoids in WS may confer stronger therapeutic effects, warranting further investigation into their specific mechanisms and applications in future studies. Amino acids and their derivatives are crucial in the physiological processes of plants, serving as the fundamental units for protein synthesis and participating in the regulation of plant metabolism [16]. The increased content of amino acids in WS may indicate its advantages in responding to environmental stress and regulating growth. This insight provides a new perspective on the cultivation and utilization of SH, suggesting that in wild environments, SH may enhance its adaptability by accumulating more amino acids, thereby improving its survival and reproductive capacity. The notable presence of lipids and alkaloids in DS and MS may be linked to their growth environments and cultivation methods, both of which considerably affect plant growth and stress resistance. Future studies should focus on optimizing cultivation techniques and environmental conditions to enhance the overall diversity of SH metabolites, thereby maximizing their medicinal value. Comparative studies on the accumulation characteristics of metabolites under different cultivation methods will provide a scientific basis for the management and utilization of SH.

**Figure 3:**
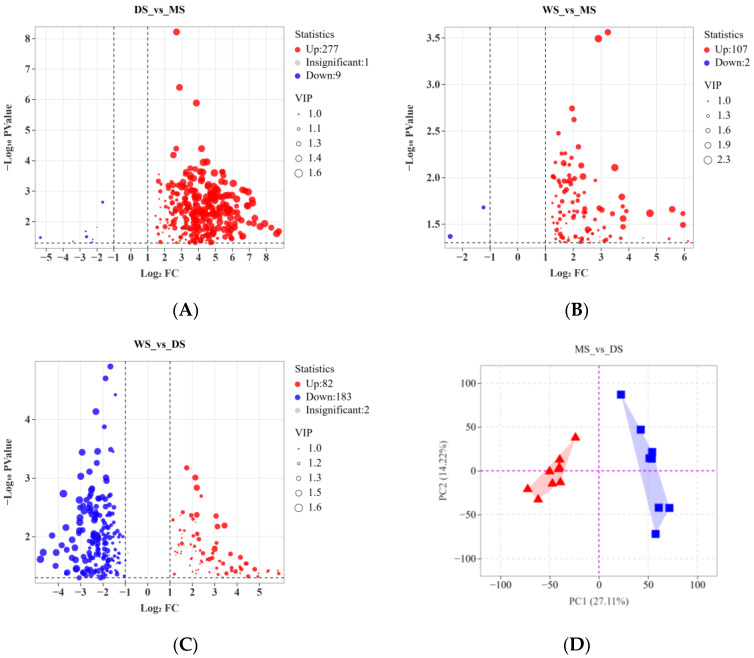
Pairwise comparative analysis of differential metabolites among WS, DS, and MS. (**A**) Volcano plot for “DS_vs_MS”; (**B**) Volcano plot for “WS_vs_MS”; (**C**) Volcano plot for “WS_vs_DS”; (**D**) PCA plot for “MS” (▲) and “DS” (■).

According to Figure 3D, the distribution of various secondary metabolites in MS is more dispersed than in DS. To investigate the influence of different cultivation methods on artificial SH in depth, this study conducted a pairwise analysis of 28 samples using OPLS-DA. The results indicated that the OPLS-DA model for “DS vs. MS” exhibited excellent goodness of fit (Q2 > 0.93, R2Y = 1, *p* < 0.05). In the comparisons of “WS vs. MS,” “DS vs. MS,” and “WS vs. DS,” we discovered 157, 109, and 267 differential metabolites, respectively, as shown in the corresponding volcano plots in Figure 3A–C. The analysis results reveal that, in the comparisons of “DS vs. MS” and “WS vs. MS,” the number of compounds with upregulated expression considerably exceeds those that are downregulated. This finding suggests that the growth modes of WS and DS substantially promote the synthesis and accumulation of secondary metabolites in SH. In the comparison of “WS vs. DS,” the downregulation of secondary metabolites in WS is particularly pronounced, further confirming the notable advantage of Duanmu cultivation of SH in metabolic product synthesis. Different cultivation methods exhibit considerable differences in their effects on SH metabolites, with Duanmu cultivation demonstrating notable superiority. These findings underscore the profound impact of cultivation methods on the synthesis of SH metabolites, particularly highlighting the advantages of Duanmu cultivation that warrant further exploration. It can be speculated that DS provides a more stable supply of nutrients and an enhanced growth environment, potentially increasing the activity of SH in secondary metabolic pathways. Additionally, future studies could focus on the functions of metabolites under varying cultivation conditions, investigating their potential roles in defense mechanisms and medicinal value, thereby offering new perspectives for the industrial application of SH.

### 2.3. Amino Acid Experiment Analysis

The analysis results indicated substantial differences in the compositions of 16 amino acids between WS and CS (Table 1). However, methionine and tryptophan were not detected. Aspartic acid was identified as the amino acid with the highest content in Sanghuang, measuring 5.50 ± 0.15 mg, which accounted for 11.32% of the total amino acids.

WS, DS, and MS were found to contain a total of 16 amino acids. The average total amino acid content of WS was 43.2 mg/g, with EAA averaging 17.71 mg/g, which accounted for 40.99% of the total amino acids. For DS, the average total amino acid content was 40.6 mg/g, with EAAs averaging 16.47 mg/g, representing 40.56% of the total amino acids. In MS, the average total amino acid content was 33.5 mg/g, with EAAs averaging 13.91 mg/g, accounting for 41.52% of the total amino acids. The ratios of EAA to non-essential amino acids for the three types of SH were 86.82, 84.54, and 84.77%, respectively. Among these, MS exhibited the highest proportion of EAA, followed by WS, and then DS. Although the amino acid profiles of the three SH samples were similar, considerable differences were observed in their total amino acid content and the composition of various amino acids, ranked in descending order as WS, DS, and MS, with WS containing 1.29 times the total amino acid content of MS. Furthermore, the total amino acid content of DS was 1.21 times that of MS, indicating variations in nutritional composition among the different CS.

### 2.4. Evaluation of the Characteristics of Three Types of SH Amino Acids

According to the ideal protein pattern spectrum of EAA proposed by the FAO and WHO revised in 1973, the ratios of EAA and the coefficient of EAA ratios (RC) of the samples are calculated (Table 2). By comparing the proportion of amino acids in food with the ideal proportion of amino acids required by the human body, it is possible to effectively evaluate the nutritional balance of amino acids in food.

According to the WHO/FAO scoring system, the EAA content of three types of SH has been evaluated, and the results are summarized in Table 3. The AAS reflects the percentage of specific amino acids in the WHO/FAO standard pattern of free amino acids. Analysis of the AAS for the three types of SH revealed significant differences (*p* < 0.05). Among these types, phenylalanine and tyrosine exhibited the highest AAS scores. Notably, only WS had AAS scores for phenylalanine and tyrosine exceeding 10 (10.42), whereas the AAS for all other SH was below 10, indicating a relatively insufficient total absolute amino acid content in the three types of SH. Although the absolute content of amino acids does not fully capture the overall nutritional value of food, the balance of amino acids is fundamentally based on the proportion of free amino acids required by the human body [17]. The amino acid Ratio Coefficient (RC) is employed to evaluate the contribution of amino acids to this balance. An RC value closer to 1 indicates that the amino acid composition of the food aligns with the WHO/FAO standard pattern [18]. Conversely, an RC value greater than one signifies a relative excess of amino acid, while an RC value less than one indicates a relative deficiency. The amino acid with the lowest RC score is identified as the first limiting amino acid in the food [19].

Combining the analyses of Table 3, the relative contribution (RC) score of methionine and cysteine (Met + Cys) in the MS group is the lowest at 0.36, identifying it as the first limiting amino acid. The RC scores of other amino acids range from 0.8 to 0.9, which do not disrupt the amino acid balance. In the DS group, Lys is identified as the first limiting amino acid, with all other amino acids exhibiting RC scores above one, except for Met + Cys, which have an RC score of 1.32. The RC scores of the remaining amino acids range from 1.00 to 1.07, maintaining an amino acid balance. In the WS group, valine is recognized as the first limiting amino acid, with the RC score for Met + Cys at 1.32, while the RC scores for other amino acids range from 1.09 to 1.13. Compared to the other two cultivation methods, WS demonstrates the highest and least variable RC score (with a score difference of 0.04 for WS, 0.32 for DS, and 0.56 for MS), indicating an optimal amino acid balance. Furthermore, all amino acid RC scores in the DS and WS groups exceed one, indicating a relative excess of amino acids, whereas all amino acid RC scores in the MS group fall below 1, indicating a relative deficiency.

The amino acid ratio coefficient method (SRC) is widely used to evaluate the nutritional value of proteins, thereby facilitating the assessment of various protein sources [20]. When assessing protein nutritional value, it is crucial to consider the biological utilization of proteins, which refers to the body’s ability to absorb and utilize these nutrients [21]. This capability is closely linked to the proportion and quantity of amino acids present in the proteins. Generally, animal proteins demonstrate higher biological utilization compared to plant proteins. In terms of nutritional value, the SRC scores for three different cultivation methods of SH are ranked as follows: WS (94.41) > DS (79.62) > MS (31.35). This ranking indicates that the amino acid content and ratio in WS most effectively meet the body’s requirements. According to the IOM model rating criteria, the IOM ratings for WS, DS, and MS are relatively low. Specifically, the IOM ratings for serine, isoleucine, phenylalanine, and tyrosine in DS and WS meet the criteria of the model spectrum, whereas MS lacks the necessary amino acid types to satisfy the IOM model ratings. Overall, WS provides the best proportion and highest quality of EAA, followed by DS, with MS receiving the lowest overall rating.

Therefore, it can be inferred that future studies on SH cultivation should prioritize enhancing the nutritional content of MS by exploring improvements in culture media and cultivation conditions to increase its amino acid richness. Additionally, incorporating molecular biology methods to analyze the mechanisms by which different cultivation techniques impact the quality of SH will provide a scientific basis for optimizing its cultivation practices.

### 2.5. Correlation Analysis of Amino Acids and Their Derivatives in SH

Based on the aforementioned findings, the analysis from the perspectives of amino acids and metabolites is as follows. (1). Amino Acid Content Analysis: WS exhibits the highest amino acid content, indicating a notable nutritional advantage. DS ranks second in amino acid content, which, while lower than WS, demonstrates substantial nutritional value. Conversely, MS displays relatively low amino acid content, potentially compromising its competitiveness in nutritional supplementation. In terms of amino acid balance, WS not only possesses the highest amino acid content but also achieves the best balance, indicating a more comprehensive amino acid profile that better meets the requirements of organisms. Although DS has a lower balance compared to WS, it presents a commendable amino acid composition. Due to its diminished amino acid content, the balance and overall nutritional value of MS may be adversely affected. (2). Metabolite Analysis: The variety and content of secondary metabolites in DS surpass those in WS, highlighting its advantages in metabolic potential and biological activity, and suggesting a potentially greater physiological or medicinal value. Although WS contains fewer types and lower quantities of secondary metabolites than DS, it remains superior to MS, indicating certain advantages in specific bioactive components. The variety and content of secondary metabolites in MS are the lowest, which may restrict its application in biological activity.

**Figure 4:**
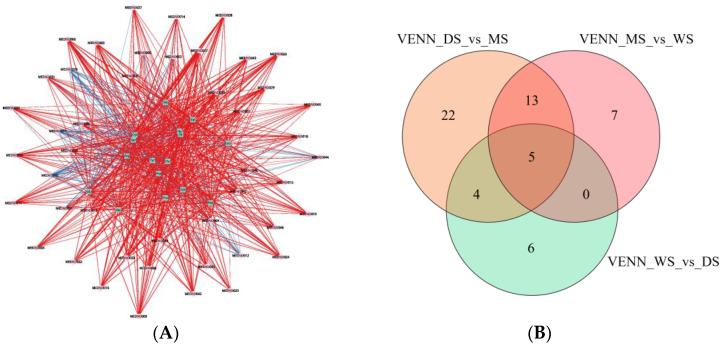
(**A**) Single-omic correlation network of differential amino acid derivatives in WS, DS, and MS; (**B**) VENN diagram of differential amino acid derivatives in WS, DS, and MS.

Through the analysis from the perspectives of amino acids and metabolites, WS has notable advantages in terms of nutritional value and metabolic potential. Although slightly inferior to WS, DS still performs well, while MS is relatively weaker in both aspects. These results provide important references for further research and applications, especially in the selection of appropriate samples for nutritional supplementation or the extraction of bioactive ingredients. To explore the relationship between secondary metabolites of SH and amino acids, metabolomics correlation analysis was conducted on 18 amino acids and 79 amino acid derivatives of WS, DS, and MS, forming a metabolomics correlation network diagram as shown in Figure 4A.

In the study on the negative ion models of WS, DS, and MS, 79 amino acid derivatives were identified, encompassing various amino acids and their derivatives. The diversity of these metabolites offers profound insights into the metabolic adaptability of SH under different cultivation conditions. Specifically, the 79 amino acid derivatives include: α-amino acids, which are fundamental components involved in protein synthesis [22]; L-cysteine-S-conjugates, associated with antioxidant capacity and potentially influencing the antioxidant properties of SH [23]; 2-(hydroxyphenyl)acetic acid, participating in metabolic pathways that affect the physiological functions of SH [24]; and N-acyl-α-amino acids, which play major roles in regulating cell signaling pathways and metabolism [25]. Additionally, compounds such as benzoyl amide, phenylalanine and its derivatives, tyrosine and its derivatives, asparagine, and glutamate are all crucial for plant growth, development, and responses to environmental stresses.

By analyzing the Venn diagram (Figure 4B), we identified 35 considerably different amino acid derivatives between WS and CS, as well as 44 considerably different amino acid derivatives produced under two distinct cultivation methods. The presence of these varying metabolites reflects the metabolic adaptability of SH to different cultivation conditions and may influence its medicinal value and nutritional composition. This suggests that different cultivation environments can lead to substantial alterations in the composition and physiological functions of SH. Furthermore, it is important to consider that the differences in the types and quantities of amino acid derivatives produced by SH in varying environments may have differing impacts on its medicinal effects. For instance, certain specific amino acid derivatives may exhibit stronger medicinal properties, and under particular conditions, their production may be inhibited, thereby affecting the overall medicinal value of SH. Therefore, when investigating the medicinal value of SH, it is essential to simultaneously consider its production environment and physiological functions to optimize its medicinal value and economic benefits.

### 2.6. Formatting of Mathematical Components

Amino Acid Score (AAS) = [Content of a specific amino acid per gram of protein to be evaluated (mg)/Content of that amino acid per gram of reference protein (mg)] × 100%.(1)

Relative amino acid score (RC) = [AAS_x_]/[Average AA_S_]; (2)

International Nutrition Organization Score (IOM) = [amino acid content per gram of protein to be evaluated (mg)/amino acid content per gram in the IOM scoring system (mg)] × 100%;(3)

(4) $$SRC=\frac{\sqrt{\sum i=1,n(RC-RC\bar{x})/(n-1)}}{RC\bar{x}}\times 100;$$

A_x_ is the content of a certain essential amino acid in the test protein; A_s_ is the content of the corresponding essential amino acid in the WHO/FAO scoring system; n is the number of essential amino acids involved in the scoring; $RC\bar{x}$ is the average value of the RC scores of amino acids in the calculation.

## 3. Discussion

Integrated analysis of amino acid and metabolomic profiles in SH samples revealed deeper insights into the mechanisms by which cultivation environments affect quality. Regarding amino acids, WS exhibited superior balance and richness compared to CS. This aligns with observations in *Ophiocordyceps sinensis* and *Cordyceps militaris* by J. Fungi [25] and Yun tao Liu [26], collectively demonstrating the critical regulatory roles of natural ecological factors—including soil [27], climate conditions, and microbial diversity [28]—in primary metabolism such as amino acid synthesis. However, while DS showed lower total amino acid content than WS, it displayed unique advantages in secondary metabolite richness. DS accumulated numerous alkaloids, flavonoids, and polyphenols with significant antioxidant, antimicrobial, and anticancer potential. This consistency with Julian Preiner’s findings on rhizobial symbiosis, enhancing both amino acid and secondary metabolite (e.g., flavonoids) biosynthesis in tungsten-stressed soybean [29] indicates that DS strategies may regulate secondary metabolic pathways to target specific bioactive compounds. Notably, our metabolomics further revealed significant enrichment in [madasiatic acid, alphitolic acid] in DS samples, corroborating Dongxue Zhang’s SH research, where plant growth regulators (e.g., paclobutrazol, PBZ) modulated triterpenoid synthesis [30]. This underscores the specific induction of functional components under DS conditions.

In omics correlation analyses, significant interactions were observed between amino acids (and their derivatives) and secondary metabolites in SH. Correlation-based network visualizations clearly delineated associations between specific amino acids and secondary metabolites, offering critical insights into SH’s metabolic regulation. These relationships may inform future breeding and cultivation strategies. Furthermore, the OPLS-DA model identified 79 statistically significant amino acids and derivatives, robustly elucidating metabolic differences across SH samples. Notably, 16 amino acids exhibited differential abundance between wild-type and cultivated samples, highlighting the substantial impact of artificial cultivation conditions on SH’s compositional profile. This underscores the necessity of optimizing growth conditions during cultivation to enhance both nutritional value and bioactivity.

Therefore, when evaluating the quality of SH under different cultivation modes, it is essential to move beyond singular metrics like amino acid content and instead adopt a comprehensive assessment of secondary metabolite diversity and bioactivity. Our study reveals that distinct cultivation modes (WS vs. CS) may shape divergent quality profiles: WS mode favors enhanced nutritional value primarily characterized by amino acids, while CS mode likely prioritizes the optimization of medicinal value driven by specific secondary metabolites. This mode-dependent quality divergence pattern may differ from observations in related species, underscoring the importance of evaluations tailored to SH’s intrinsic characteristics. These findings deepen the understanding of how cultivation environments shape SH quality and provide a theoretical foundation for future targeted improvements. Strategies include simulating key ecological factors to elevate amino acid levels in WS or modulating stress conditions to induce the accumulation of target secondary metabolites in CS. Given SH’s dual value as food and medicine, its rich composition—encompassing amino acids, flavonoids, polysaccharides, and other bioactive compounds—holds significant potential both as functional food additives or nutritional supplements leveraging WS’s nutritional advantages and in developing pharmaceuticals or health products with specific health benefits capitalizing on CS’s bioactive components.

## 4. Materials and Methods

### 4.1. Instruments

Mettler Toledo one-in-a-million electronic balance, XP26 one-in-a-million analytical balance, Mettler Toledo one-in-ten-thousand electronic balance, and ML204 one-in-ten-thousand analytical balance (Mettler-Toledo Instrument Co., Ltd., Shanghai, China); Advantage A10 Milli-Q ultrapure water system (Millipore Corporation, Burlington, MA, USA); Elmasonic S30 (H) ultrasonic cleaner (Dexiang Technology Co., Ltd. Hamburg, Germany); 116B swing-type high-speed Chinese medicine grinder (Yongli Pharmaceutical Machinery Co., Ltd., Changzhou, China); and ACQUITY UPLCTM ultra-high performance liquid chromatograph and Xevo G2 Q-Tof mass spectrometer (Waters Corporation, Parsippany, NJ, USA) were used to obtain measurements.

### 4.2. Sample Collection

The WS and two CS samples were collected by the research team during surveys conducted in various locations. All samples consisted of dried fruiting bodies of the fungus *Sanghuangporus vaninii* (Ljub.), which belongs to the family Polyporaceae. Detailed sample information is provided in Table 4.

### 4.3. Reagents

Methanol and acetonitrile were purchased from Merck Life Science Co., Ltd. in Shanghai, China. Acetonitrile (lot No. 207866) was purchased from Fisher Scientific in the Hampton, NH, USA. Formic acid (lot No. C12122684) was purchased from Shanghai McLean Biochemical Technology Co., Ltd. Phosphomolybdic acid reagent was purchased from Beijing Huake Sheng Fine Chemical Trading Co., Ltd. Other analytical grade reagents were purchased from China National Pharmaceutical Group Chemical Reagent Co., Ltd. (Shanghai, China).

### 4.4. Sample Preparation

#### 4.4.1. Preparation of Secondary Metabolites Solution

Sanghuang powder (prepared by pulverizing the raw material and passing through a No. 4 sieve; particle size ≤ 250 μm) (0.5 g) was accurately weighed. Then 20 mL of 70% methanol was added, and the mixture was sonicated for 1 h, followed by filtration. The filtrate was concentrated to approximately 2 mL, passed through a 0.22 μm membrane filter, and stored for future use.

#### 4.4.2. Preparation of Total Amino Acid Solution

The powder of SH (50 mg) was accurately weighed and placed in a test tube measuring 180 mm × 18 mm. The tube was sealed under vacuum and hydrolyzed at 110 °C in an oven for 24 h, after 6 mL of 6 mol/L hydrochloric acid was added. After the hydrolysis, the seal was opened, and the contents were transferred to an evaporating dish. The mixture was dried, and the residue was washed in portions with 0.02 mol/L hydrochloric acid solution. The washing solution was combined and filtered, and the filtrate was transferred to a 5 mL volumetric flask. It was then diluted with 0.02 mol/L hydrochloric acid solution to the mark.

### 4.5. Fully Automatic Amino Acid Analyzer Resolution and LC-Q-TOF-MS Analysis

#### 4.5.1. Chromatographic Conditions

The experiment employed an ACQUITY UPLC BEH C18 column (2.1 × 100 mm, 1.7 μm). The mobile phase was a composition of 0.1% formic acid solution (A) and acetonitrile (B). The column temperature was maintained at 30 °C, and the injection volume was set at 5 μL. The flow rate was controlled at 0.3 mL/min, and the gradient elution program is detailed in Table 5.

#### 4.5.2. Mass Spectrometry Conditions

The electrospray ionization source operated in the MSE continuum mode, employing negative ion detection. The full scan range was set from 100 to 1500 *m*/*z*. Parameters included a capillary voltage of 3.0 kV, cone voltage of 30 V, cone gas flow rate of 50 L/h, desolvation gas nitrogen flow rate of 600 L/h, desolvation gas temperature of 300 °C, an argon flow rate of 0.15 mL/min, and an ion source temperature of 120 °C. Real-time calibration was conducted using leucine enkephalin throughout the acquisition process.

### 4.6. Statistical Analysis of SH Secondary Metabolites Data

The metabolomics data obtained from SH were processed for peak extraction and normalization using Msdial.v5.4 software. The metabolomics data of SH were compared with and referenced against the Public EXP, PlaSMA, MetaboBASE, Public, and Mass Bank databases, as well as relevant literature. A total of 749 compounds were identified in the metabolomics data of SH.

Multivariate statistical analysis was performed using the MetaboAnalyst platform. The multivariate statistical methods employed included Principal Component Analysis (PCA), Hierarchical Clustering Analysis (HCA), and Orthogonal Partial Least Squares Discriminant Analysis (OPLS-DA). In this study, PCA and HCA were applied for metabolomic data analysis of all SH samples. PCA and HCA analyses were conducted for all samples to screen for differential metabolites in paired combinations. In the comparison process, OPLS-DA was used, and the stability of the log2-transformed PLS-DA model was validated through 200 permutation tests.

### 4.7. Evaluation Indicators for the Amino Acid Content of SH

The multivariate indices used to characterize amino acids in SH include the Amino Acid Score (AAS). This score is calculated by comparing the content of a specific essential amino acid (EAA) per gram of protein in the tested food with the corresponding amino acid content per gram of protein in the World Health Organization (WHO)/Food and Agriculture Organization (FAO) pattern spectra, which were published jointly by the WHO, FAO, and United Nations University. A higher AAS indicates a greater nutritional value of the protein. The Institute of Medicine (IOM) score refers to the guidelines established by the IOM. The Ratio of Content (RC) and the Standardized Ratio of Content (SRC) scores employ the amino acid ratio method, which assesses the nutritional value of protein by comparing the content and requirements of various amino acids. The eight commonly used EAA are tryptophan, lysine, phenylalanine, isoleucine, leucine, methionine, threonine, and cysteine. These amino acids cannot be synthesized by the human body and must be obtained through diet; thus, their content and proportions considerably influence the nutritional value of protein.

## 5. Conclusions

Different cultivation methods have a notable impact on the nutritional composition and functional properties of SH, which suggests that the source and cultivation methods should be considered when developing related foods. WS may have advantages in food development due to its rich amino acids and flavonoids, while DS and MS may have advantages in some components. Their overall balance and diversity of bioactive ingredients may not be as good as WS. Therefore, selecting the appropriate cultivation method and harvest time can help maximize the retention of the nutritional value of SH and ensure the health benefits of food. In conclusion, SH not only demonstrates extensive application prospects in the medicinal field but also shows its unique value in the food field. Through in-depth research and development of SH, a scientific basis can be provided for its application in the food industry, while promoting the dissemination and implementation of healthy dietary concepts. Future research can focus on food processing technology of SH, development of functional foods, and clinical applications of bioactive components, to fully leverage the medicinal and edible properties of SH for the benefit of human health.

## Figures and Tables

**Table 1:** Amino acid content of Sanghuang in three different cultivation methods (mg/g). Values are expressed as mean ± SD (*n* = 9).

Amino Acid		Strains		
		MS	DS	WS
**EAA**	Thr	2.08 ± 0.07	2.62 ± 0.05	2.84 ± 0.07
	Val	2.34 ± 0.07	2.84 ± 0.05	2.95 ± 0.07
	Met	ND	ND	ND
	Try	ND	ND	ND
	Ile	2.21 ± 0.06	2.7 ± 0.04	2.82 ± 0.07
	Leu	3.5 ± 0.11	4.06 ± 0.08	4.41 ± 0.11
	Phe	1.98 ± 0.07	2.24 ± 0.05	2.49 ± 0.07
	Lys	1.8 ± 0.07	2.01 ± 0.04	2.2 ± 0.09
**NEAA**	Tyr	1.73 ± 0.07	1.61 ± 0.03	1.85 ± 0.05
	Glu	2.79 ± 0.1	3.96 ± 0.07	4.05 ± 0.1
	Ser	3.04 ± 0.12	3.54 ± 0.07	3.75 ± 0.13
	Gly	1.86 ± 0.07	2.24 ± 0.05	2.52 ± 0.08
	Ala	2.2 ± 0.08	2.71 ± 0.05	2.85 ± 0.08
	Cys	0.03 ± 0.01	0.11 ± 0.01	0.11 ± 0.01
	Asp	3.73 ± 0.14	4.53 ± 0.08	4.93 ± 0.14
	His	0.64 ± 0.03	0.78 ± 0.02	0.83 ± 0.03
	Arg	1.9 ± 0.07	2.24 ± 0.05	2.25 ± 0.09
	Pro	1.73 ± 0.06	2.39 ± 0.04	2.34 ± 0.07
**TAA** **TEAA** **TNEAA**		33.5 ± 0.06	40.6 ± 0.04	43.2 ± 0.07
		13.91 ± 0.07	16.47 ± 0.06	17.71 ± 0.08
		16.02 ± 0.06	19.48 ± 0.05	20.89 ± 0.07

**Table 2:** Amino acid content defined by WHO/FAO and IOM models (mg/g). Values are expressed as mean ± SD (*n* = 9).

Amino Acid	Strains			WHO/FAO (mg/g)	IOM (mg/g)
	MS	DS	WS		
His	0.64 ± 0.03	0.78 ± 0.02	0.83 ± 0.03	15	17
Thr	2.08 ± 0.07	2.62 ± 0.05	2.84 ± 0.07	40	27
Lys	1.8 ± 0.07	2.01 ± 0.04	2.2 ± 0.09	55	51
Leu	3.5 ± 0.11	4.06 ± 0.08	4.41 ± 0.11	70	55
Ile	2.21 ± 0.06	2.7 ± 0.04	2.82 ± 0.07	40	25
Met + Cys	0.03 ± 0.01	0.11 ± 0.01	0.11 ± 0.01	35	25
Phe + Tyr	4.59 ± 0.17	5.97 ± 0.11	6.25 ± 0.19	60	47
Val	2.34 ± 0.07	2.84 ± 0.05	2.95 ± 0.07	50	32

**Table 3:** Analysis of amino acid characteristics of Sanghuang under three different cultivation methods. Values are expressed as mean ± SD (n = 9).

Amino Acid	Amino Acid Score (AAS, %)			Ratio Coefficient (RC, %)			Ratio Coefficient (RC, %)			Score of RC (SRC, %)		
	MS	DS	WS	MS	DS	WS	MS	DS	WS	MS	DS	WS
His	4.27	5.22	5.53	0.85	1.04	1.11	37.65	45.88	48.82	31.35	79.62	94.41
Thr	5.24	6.55	7.11	0.83	1.04	1.13	77.04	97.04	105.19			
Lys	3.27	3.65	4.08	0.93	1.01	1.10	35.29	39.41	43.14			
Leu	5.05	5.81	6.30	0.88	1.02	1.11	63.64	73.82	80.18			
Ile	5.53	6.75	7.05	0.86	1.05	1.09	88.43	108.02	112.82			
Met + Cys	0.09	0.31	0.31	0.36	1.32	1.32	1.23	4.41	4.42			
Phe + Tyr	7.65	9.95	10.42	0.82	1.07	1.12	97.66	127.02	132.98			
Val	4.68	5.68	5.92	0.86	1.05	1.09	73.13	88.75	92.19			

**Table 4:** Information Table of Sanghuang Samples.

Number	Batch Number	Latitude (°N)	Longitude (°E)	Place of Origin	Collection Time
WS01	SH20181201	29.565583	94.304296	Zayul, Tibet	01122018
WS02	SH20191201	29.565583	94.304296	Zayul, Tibet	01122019
WS03	SH20191202	29.565583	94.304296	Zayul, Tibet	02122019
WS04	SH20201201	29.565583	94.304296	Zayul, Tibet	01122020
WS05	SH20201202	29.565583	94.304296	Zayul, Tibet	02122020
WS06	SH20181101	29.565583	94.304296	Zayul, Tibet	01112018
WS07	SH20181202	29.565583	94.304296	Zayul, Tibet	02122018
WS08	SH20180602	29.565583	94.304296	Zayul, Tibet	02062018
WS09	SH20191203	29.565583	94.304296	Zayul, Tibet	03122019
WS10	SH20191204	29.565583	94.304296	Zayul, Tibet	04122019
DS01	SH20201203	115.704881	36.838277	Linqing, Shandong	03122020
DS02	SH20191205	115.704881	36.838277	Linqing, Shandong	05122019
DS03	SH20201204	115.704881	36.838277	Linqing, Shandong	04122020
DS04	SH20201206	115.704881	36.838277	Linqing, Shandong	06122020
DS05	SH20201207	115.704881	36.838277	Linqing, Shandong	07122020
DS06	SH20191201	115.704881	36.838277	Linqing, Shandong	01122019
DS07	SH20191202	115.704881	36.838277	Linqing, Shandong	02122019
DS08	SH20201201	115.704881	36.838277	Linqing, Shandong	01122020
DS09	SH20201202	115.704881	36.838277	Linqing, Shandong	02122020
MS01	SH20181101	31.454120	115.567047	Jinzhai, Anhui	01112018
MS02	SH20181202	31.454120	115.567047	Jinzhai, Anhui	02122018
MS03	SH20180602	31.454120	115.567047	Jinzhai, Anhui	02062018
MS04	SH20191203	31.454120	115.567047	Jinzhai, Anhui	03122019
MS05	SH20191204	31.454120	115.567047	Jinzhai, Anhui	04122019
MS06	SH20201203	31.454120	115.567047	Jinzhai, Anhui	03122020
MS07	SH20191204	31.454120	115.567047	Jinzhai, Anhui	04122019
MS08	SH20201203	31.454120	115.567047	Jinzhai, Anhui	03122020
MS09	SH20191205	31.454120	115.567047	Jinzhai, Anhui	05122019

**Table 5:** Gradient elution program for ultra-high performance liquid chromatography.

Time (min)	Mobile Phase A (%)	Mobile Phase B (%)
0~6	95→88	5→12
6~16	88→85	12→15
16~20	85→75	15→25
20~25	75→50	25→50
25~31	50→33	50→67
31~37	33→25	67→75
37~39	25→20	75→80

## Data Availability

The data supporting this study have been deposited in a public repository: Experimental raw data: Repository: Figshare DOI: 10.6084/m9.figshare.29436071.

## Abbreviations

The following abbreviations are used in this manuscript:

SH	SH
WS	Wild SH
DS	Duanmu SH
MS	Mycelium SH
CS	Cultivated SH
